# What every intensivist should know about handovers in the intensive care unit

**DOI:** 10.5935/0103-507X.20170020

**Published:** 2017

**Authors:** Pablo Perez D'Empaire, Andre Carlos Kajdacsy-Balla Amaral

**Affiliations:** 1 Department of Anesthesia, University of Toronto - Toronto, Ontário, Canadá.; 2 Critical Care Medicine, Sunnybrook Health Sciences Centre - Toronto, Ontário, Canadá.; 3 Interdepartmental Division of Critical Care Medicine, University of Toronto - Toronto, Ontário, Canadá.

## BACKGROUND

Handover, the act of transferring information and accountability between clinicians, is recognized by the World Health Organization^(1)^ and critical care societies^(2)^ as one of the key elements of quality and safety. With changes in residents' working hours in the past years in the United States,^(3)^ the number of handovers increased considerably, and a vast body of literature now exists for both critical care and postoperative patients.^(4)^ Poor communication during handovers is associated with an increase in medical errors and adverse events,^(5-7)^ and several tools and interventions exist to improve communication and reduce medical errors.^(8)^ Critical care units and postoperative recovery units are strategic areas where patients are more vulnerable to communication breakdowns, given the complexity of these areas and the multiple team transitions that occur during patient care.^(5)^

## WHAT IS A HANDOVER?

The current literature provides different definitions of handovers depending on the scope of the area or the type of communication; however, the definition by Cohen et al. in a recent literature review^(9)^ ("the exchange between health professionals of information about a patient, accompanying either a transfer of control over, or of responsibility for, the patient") captures the essential elements of communication during the transitions of care for patients. This means that a handover can occur when patients are changing teams (or control, for example, when they come into the intensive care unit [ICU] from the operating room) or when shifts are changing (responsibility is changing, for example, when the night team takes over for patients in the ICU).

## CHALLENGES TO HANDOVERS IN CRITICALLY ILL PATIENTS

Critically ill patients undergo multiple changes in teams during their care, with problems in communication at every step of these transitions, including admission from the operating room,^(10)^ ICU stay,^(11)^ ICU transfers to the ward and transfers between different ICUs.^(7)^ These can be errors of omission or corruption of information,^(12)^ impact clinical decision making^(13)^ and discharge planning.^(7)^

Human factors and organizational aspects of the environment play an important role in facilitating or mitigating these errors. For example, errors of omission may occur due to distractions during handovers (such as other team members asking for directions on non-urgent aspects of care), disorganized information (such as critical blood work or vital signs that are not readily available for discussion), and reliance on memory.^(14)^ The corruption of information may occur due to poor construction of the message (e.g., use of jargon, inaccurate word choice) or due to cognitive biases, such as when patients have an unclear diagnosis and are described as having an established diagnosis during handover.

In many situations, the conversation on handovers is unidirectional, in which the person handing over the patient describes the clinical situation and the current treatments. However, in complex patients with many diagnoses and clinical uncertainties, simple one-way communication may not be enough. Even with the accurate information and proper language for an adequate handover process, it may not be possible to provide a full comprehension of the most important and uncertain aspects of a patient's clinical course. In these situations, two-way communication with both parties, discussing the diagnosis and treatments from different perspectives, allows for a new construction of the clinical scenario, which may have a positive impact on the communication process.^(15)^ In a recent study of cross-covering nighttime clinicians, when patients were cared for at night by an incoming clinician that did not participate in their care during the day, they were more likely to have more diagnostic tests and changes in treatment overnight, and they had a lower mortality. These data suggest that the incoming clinician's different perspective may have helped them identify the problems that were overlooked by the daytime clinicians.^(13)^ Once we acknowledge this crucial function of re-thinking about the patient during handovers, it is clear that we need to focus not only on what information is communicated but also on the interactions between clinicians during a handover.

In the ICU setting, there are several barriers that impact the effectiveness and safety of the handover (Table 1).

**Table 1:** Barriers to effective and safe handovers

Standardization	Lack of formal handover education Staff resistant to changes in handover process Lack of handover protocols Lack of electronic tools to support handover
Organizational	Multitasking during handover Multiple interruptions and distractions Time constraints Noisy location
Communication skills	Omissions, errors, or misunderstandings Language barriers Social interactions occurring during handover Incorrect information recall Hierarchical culture that discourages questions Differences in clinical knowledge
Clinical factors	Patients with multiple medical problems Large number of patients Changes in patient status preceding handover

## WHAT CAN WE DO TO IMPROVE HANDOVERS?

### Memory aids

The most basic and efficient level of improvement is to use memory aids. These can take many forms, from a simple note-taking process during handovers to "low-tech" solutions, such as electronic documents that exist locally in the ICU computer, to more complex handover systems that integrate with electronic medical records. The basic tenet is to avoid reliance on memory. A commonly used method is to develop a handover-specific form; in a recent systematic review, this was the most commonly used intervention;^(16)^ however, the quality of the evidence of these studies is limited.

### Standardization of handovers

Although strategies with mnemonics have shown mostly conflicting results or were described in studies of poor quality,^(15)^ they continue to proliferate in the handover literature; a systematic review of handover mnemonics resulted in the identification of twenty-four different mnemonics up to 2009.^(17)^ The best evidence comes from a recent before-after study of a new mnemonic (I-PASS), where the use of standardization resulted in a 23% decrease in medical errors in a pediatric population.^(8)^ Care must be taken, however, to adopt this approach, as the implementation was very complex, including several technological components, which limits the generalizability of the tool. In spite of the limited evidence to support standardization, teams should be encouraged to consider standardizing elements of handover, paying special attention to commonly missed and important information in their own settings.

### Handover protocols

Many institutions have focused on developing structured handover protocols to minimize errors, borrowing strategies from the automotive industry, such as Six-Sigma, or from Formula-One to improve handovers to the ICU;^(10)^ both strategies have the standardization of the processes in common, including clear roles for participants, task sequences, anticipation of events, checklists and handover-specific forms. These structured moments of handover are different from standardization as they focus not only on which elements need to be discussed but also on when and where handovers occur, who should be present, and what is the sequence of presentation, and they frequently incorporate elements that enable two-way communication in their format.

## CONCLUSIONS

Handovers are an important moment in patient safety with potential to improve quality and efficiency of care. Understanding that handovers should not be a one-way communication is crucial when caring for complex patients, such as critically ill patients. Clinicians and intensive care unit directors should consider many simple strategies that can improve communication and are unlikely to cause harm, despite limited evidence.
